# Partially occluded weed classification using vision transformers and convolutional neural networks for precision agriculture

**DOI:** 10.1038/s41598-026-59448-0

**Published:** 2026-06-23

**Authors:** Euan Hall, Emmanuel Junior Zuza, Karen Rial Lovera, Chris McCarthy

**Affiliations:** 1 School of Agricultural Science and Practice, Royal Agricultural University, Cirencester, GL7 6JS UK; 2 Zanvyl Krieger School of Arts & Sciences, Johns Hopkins University, Baltimore, MD 21218 USA

**Keywords:** Vision transformers, Convolutional neural networks, Weed classification, Occlusion, Precision agriculture, Environmental sciences, Mathematics and computing, Plant sciences

## Abstract

Automated weed detection is essential for site-specific herbicide application, that can result into the reduced environmental footprint of conventional agriculture. However, for field deployment of automated weeding devices, occlusion remains a critical challenge that can weaken the precision of weed identification. Here, we compare the performance of Vision Transformers (ViT-B16 & PvTv2) and Convolutional Neural Networks (EfficientNet-B0 & ResNet-50) in accurate weed detection, using controlled synthetic occlusion levels (0%, 25%, and 50%). We found that ViT-B16 has superior occlusion resilience, with image testing accuracy increasing from 80% to 86% under 50% occlusion. In contrast, the testing accuracy of PvTv2, EfficientNet-B0 and ResNet-50 dropped from 45 to 76% under similar conditions. Multivariable regression confirmed architecture type as the dominant testing accuracy driver (*p* ≤ 0.001), with ViTs outperforming CNNs by an average of 14.56% points. These results suggest that occlusion resilience is not uniform across architectural variants but depends critically on attention-based design. Consequently, for real time deployable automatic weed detection systems, hybrid architectures that balance ViT global context with CNN computational efficiency represent a critical future direction. Such approaches can support precise herbicide application, reduce chemical inputs, and enable more sustainable crop protection through reliable AI-driven automation.

## Introduction

Wheat provides one-fifth of the global calories consumed by humans, making weed-induced yield losses of 23–50% a major threat to food security^1,2^. Herbicides have been the dominant control strategy of weeds since the 1950s^3^, but their extensive use threatens ecosystems. Herbicide production and application generate substantial greenhouse gas emissions, while residues contaminate soils and aquatic ecosystems, with exposure linked to cancer, endocrine disruption, and neurological disorders^4,5^. Broad-spectrum applications deplete resources for farmland birds and pollinators^6^, and over 520 herbicide-resistant weed biotypes cost UK wheat producers £400 million annually^7^. These pressures require weed management strategies that reduce chemical dependence while maintaining productivity.

Precision agriculture (PA) offers sensor-guided, site-specific interventions. For example, convolutional neural networks (CNNs) enable automated weed identification, classification and targeted herbicide application^8^, however, performance degrades under field conditions with crop or soil occlusion^9^. Despite advances in PA, weed identification using deep learning remains underexplored compared to other agricultural computer vision applications.

Vision transformers (ViTs), introduced in 2020, leverage self-attention mechanisms to capture global image context and demonstrate robustness to occlusion versus CNNs^10^. ViT architectures have achieved substantial gains in crop disease detection achieving ≥ 95% accuracy in detection of wheat diseases, outperforming CNN architectures such as AlexNet^11^ and VGG16^12^ by 6.68% and 4.94% respectively. Lightweight variants like MobileViT enable field deployment^13^, and hybrid Swin Transformer approaches enhance feature extraction in complex backgrounds^14^. Despite success in disease detection^15^, ViT applications for weed identification remain limited, a critical gap given the complexity of weed-crop discrimination under heterogeneous field conditions.

CNNs struggle to capture long-range spatial dependencies necessary for distinguishing morphologically similar species under variable conditions^16^. While transfer learning approaches improve disease classification^17^, it can suffer from limited domain transferability and potential bias^18^. This is particularly problematic for weed detection and classification where within-field variability and crop-weed similarity demand robust contextual understanding.

This paper evaluates the performance of ViTs and CNN-based models in accurate weed classification within wheat fields, when presented with occluded data. We assess the effect of image resolution on classification accuracy to inform deployment strategies for real-world applications. Through these evaluations, we quantify the potential for targeted herbicide reductions and establish the potential for hybrid ViT-CNN models as pathways toward precision interventions that mitigate resistance evolution, preserve farmland biodiversity, and reduce environmental contamination.

## Methods

### Architectures

EfficientNet-B0 follows a compound scaling approach, uniformly scaling network width, depth, and resolution using mobile inverted bottleneck convolution (MBConv) blocks enhanced with squeeze-and-excitation attention^19^. This architecture emphasises parameter efficiency and baseline image feature extraction^20^. However, its dependence on local receptive fields limits its ability to recover critical weed features when part of the plant structure is hidden.

ResNet-50 uses deep residual learning architectures with identity-based skip connections, which help mitigate vanishing gradients and stabilise training of deep convolutional layers^21^. Yet it still relies on hierarchical convolutional feature extraction. In occlusion-prone settings, the lack of global context aggregation means individual weed features can be lost if partially hidden, constraining generalisation under obstruction^22^.

ViT-B16 uses a pure transformer architecture for image classification by dividing the input image into non-overlapping 16 × 16-pixel patches. Each patch is linearly embedded and processed through 12 transformer encoder layers with multi-head self-attention^23^. The global attention mechanism allows ViT-B16 to integrate spatial dependencies across the full image, enabling it to distinguish morphologically similar weeds and crops even under partial occlusion^24^.

PvTv2 extends the transformer model with a hierarchical, pyramid structure that progressively downsamples features across multiple encoder stages. It combines local embedding and multi-scale attention to capture fine-grained and contextual cues^25^. However, its reliance on structured patch merging weakens its resilience under heavy occlusion, as it retains some spatial bias from its hierarchical design^26^.

### Data collection

**Fig. 1:**
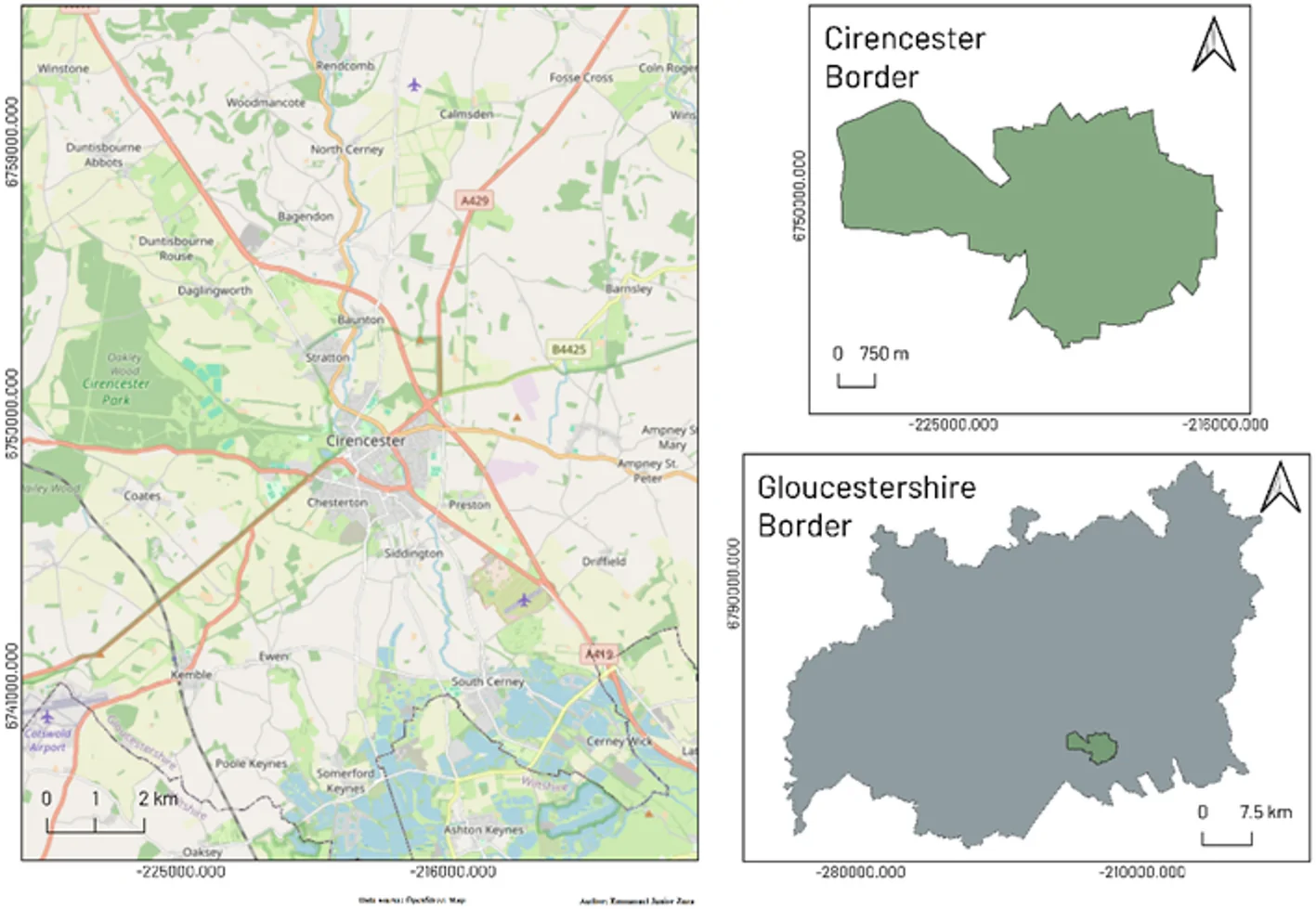
Study location.

Image data was collected at Coates Manor Farm, Cirencester (Fig. 1), From April to July 2025, using a DJI Mini 3 drone (12 MP CMOS sensor, 4:3 aspect ratio) and Google Pixel 5 smartphone (12.2 MP rear camera, handheld capture above the canopy). Images were acquired, across multiple fields, under natural daylight conditions and time-of-day windows (morning and midday) to introduce realistic illumination variability. The host crop at the study site was winter wheat (*Triticum aestivum*) at the tillering to stem elongation stages.

**Fig. 2:**
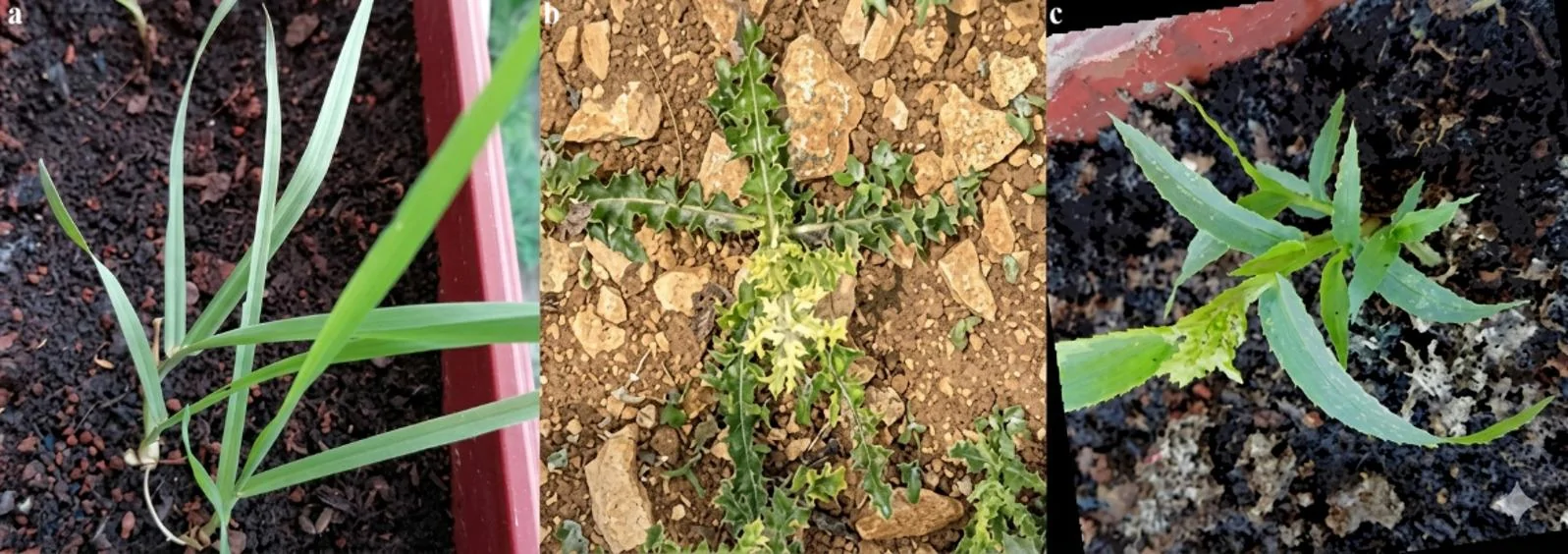
Sample images: (**a**) *Avena fatua* (**b**) *Cirsium arvense* (**c**) *Setaria viridis*.

To enhance dataset diversity and improve model robustness, we incorporated images from the publicly available dataset of Toskova and Toskov^27^, which provides field-acquired images of common arable weed species under varied background and lighting conditions. This yielded a total of 970 images across three weed species: *Avena fatua* (wild oat, 753 images), *Cirsium arvense* (creeping thistle, 118 images), and *Setaria viridis* (green foxtail, 99 images) (Fig. 2). This multi-source approach introduced variability in camera type, capture height, background complexity, and soil characteristics, factors known to influence model performance and generalisability^28^.

### Definition and quantification of occlusion

In our study, occlusion is defined as the partial obscuration of weed features within an image due to overlapping vegetation, crop canopy shadows, soil residues, debris, or adjacent plant material. Two complementary forms of occlusion were addressed in this study:

- *Naturally occurring occlusion*: Field-acquired validation images contained authentic occlusion from soil, crop canopy, and shadows. Each validation image was visually inspected and assigned a qualitative occlusion category (low, moderate, high). This reflects the reality that agricultural deployment environments are inherently unstructured, and architectures must generalise to weeds partially hidden by crop biomass, soil clods, or shadow patterns that vary diurnally and seasonally.
- *Controlled synthetic occlusion*: To enable systematic and reproducible evaluation of occlusion effects, we applied random erasing at three controlled intensities corresponding to the probability *p* that a randomly sized and positioned rectangular patch (covering 2–40% of the image area, aspect ratio 0.3–3.3) would be erased and replaced with random pixel values during training.

This dual approach is similar to methodological conventions in pest and disease identification studies^29–31^, where occlusion is acknowledged as a primary challenge for field identification and deployed computer vision systems. Consequently, by combining real field occlusion with controlled synthetic degradation, we systematically quantified the relationship between occlusion intensity and model performance while testing generalisation to actual agricultural scenarios.

### Image pre-processing and augmentation

Because CNNs perform well on smaller datasets whereas ViTs require larger training sets, we implemented Instance Discrimination with Multi-Crop and CutMix (IDMM) augmentation to mitigate data limitations and strengthen ViT performance on limited samples^32^. Field-collected images were annotated using LabelImg^33^ in PASCAL VOC format, with each weed features labelled at the species level by trained annotators. All images underwent standardised pre-processing including resizing to 224 × 224 pixels, histogram equalisation (mean and standard deviation = 0.5 across RGB channels), normalisation, and noise reduction to enhance feature clarity and to reduce data artifacts^34^.

The complete dataset was stratified by species and randomly shuffled, then partitioned into training (70%, *n* = 679) and validation (15%, *n* = 145), subsets to minimise sampling bias and preserve class proportions across splits. An independent testing (15%, *n* = 146) set containing field images with naturally varying occlusion levels, unseen during training, was used to assess model robustness under realistic field conditions where weeds were partially hidden by soil, crops, or canopy shadows.

**Fig. 3:**
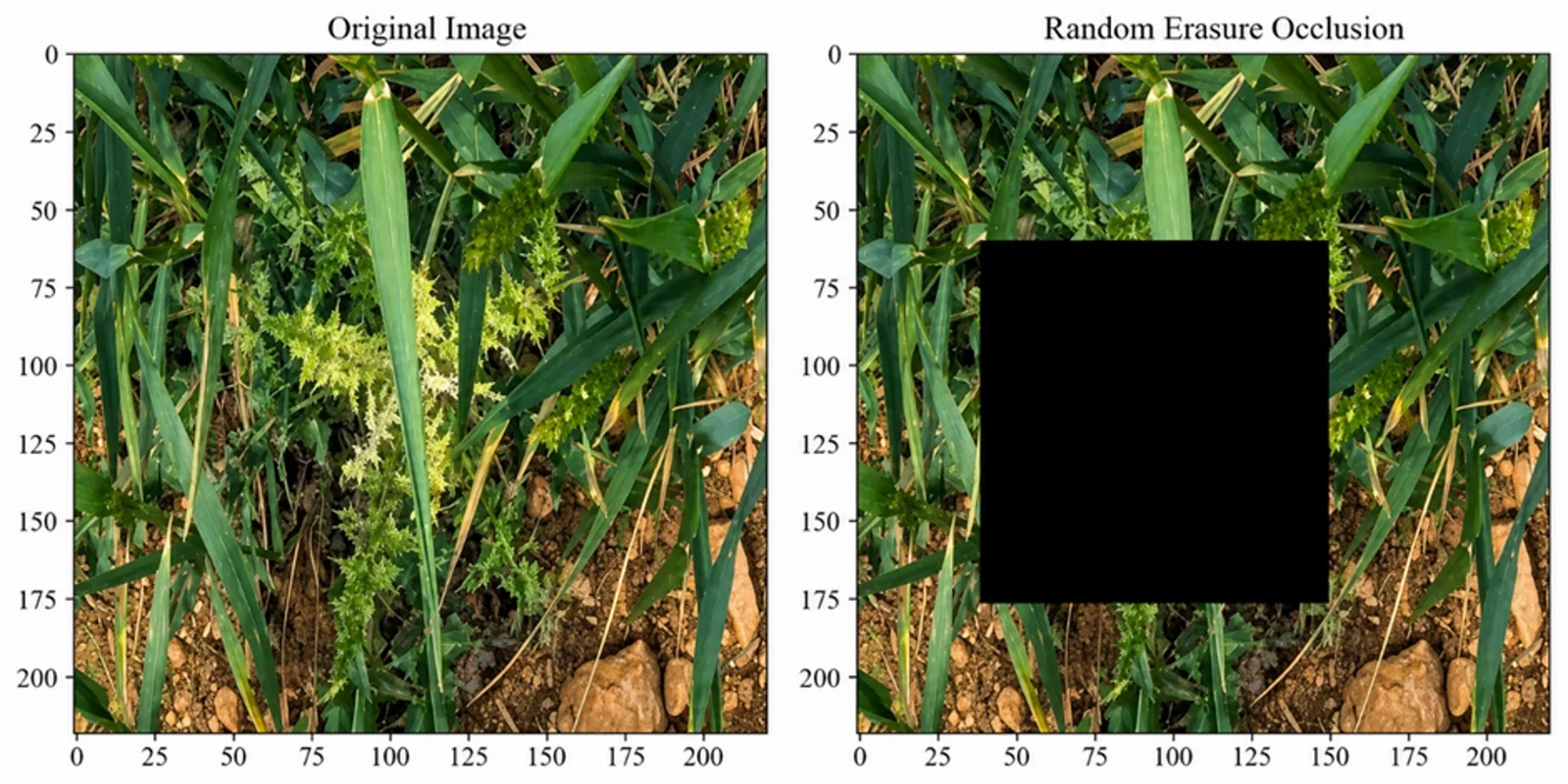
Sample of applied random erasure compared to the original image.

To simulate field heterogeneity and improve generalisation, we implemented TrivialAugmentWide^35^, applying stochastic combinations of geometric (random crops, rotations) and photometric (colour jittering) transformations (Fig. 3). Augmented datasets were pre-computed and cached to reduce training latency. RGB channels were preserved to retain spectral information critical for differentiating morphologically similar weed-crop species.

To test robustness to occlusion, we incorporated random erasing at three intensity levels (*p* = 0, 0.25, 0.5), progressively increasing the proportion of artificially occluded pixels. This controlled framework enabled systematic evaluation of performance degradation under increasing occlusion. Validation images exhibiting natural occlusion were withheld from training to test model generalisation to authentic agricultural scenarios, helping control chances of data leakage and bias during training. This precaution is particularly important given that augmentation strategies, while necessary for class balancing, can inadvertently introduce dataset contamination if validation samples leak into training batches.

We also included greater numbers of image augmentation to balance out classes as a result increased the dataset size. While this is recommended, it can however increase chances of bias and data leakage during training, affecting the overall results. This is similar to tensions between augmentation necessity and contamination risk, which is well-documented in agricultural pest and disease identification studies. This is the reason synthetic oversampling of minority classes including rare pests and pathogens must be carefully segregated from validation protocols to preserve metric integrity^31^.

### Activation functions

We applied ReLU^36^ in the PvTv2 models^32^ and SiLU^37^ in the standard ViT configurations, enabling direct comparison between sharp and smooth activation regimes under identical training and augmentation conditions. While early activation functions such as sigmoid and tanh suffered from vanishing gradients in deeper networks^38^, modern alternatives such as GeLU and SiLU offer smoother transitions and improved gradient propagation^39^. On validation data, SiLU-based ViTs showed marginally improved gradient flow and reduced training oscillations under high-occlusion scenarios.

### Optimisation

We compared four gradient-based optimisers under identical training conditions: SGD as a baseline, Adagrad to address sensitivity to class imbalance through per-parameter adaptive learning rates^40,41^, Adam for improved convergence stability^42^, and AdamW, which incorporates weight decay for stronger generalisation^41^. Optimiser selection is typically guided empirically rather than by theoretical considerations^43^, motivating the comparative evaluation reported in the results.

### Batch size and training configuration

Batch size influences gradient stability, computational efficiency, and generalisation performance through its effect on stochastic gradient estimation. Larger batches stabilise gradient updates but reduce stochasticity, potentially degrading generalisation, while smaller batches introduce variance that can improve generalization at the cost of slower convergence^44^. As a result, a batch size of 32 was selected for our study based on optimal convergence behaviour and computational efficiency. Secondly, early stopping was implemented to get the weights of the best run for each model. In the final approach we used a random seed of 42 and learning rate of 0.00001 to obtain our results. While the learning rate was low, it was observed that higher learning rates caused increased disturbances during training due to the dataset’s size.

### Model validation

To reduce class imbalance, shuffling and randomly splitting of the dataset ensured proportional representation of weed species across training (70%), validation (15%), and test (15%) subsets. However, because of our dataset accuracy alone would be a misleading metric. This is because a model can achieve high overall correctness by excelling on a dominant class while failing on rarer species, an outcome unacceptable for practical weed detection where all species require reliable identification. We, therefore, quantified architecture performance using F1-scores, calculated independently for each class and then averaged without class weighting. This approach ensured that performance of one weed species did not compensate for poor performance on another by the models. The F1-score metric is one of the commonly used in object detection and masking research domain.

We evaluated the F1-score at every epoch during training, and the model checkpoint achieving the highest validation F1-score was retained for final testing on the held-out test set containing naturally occluded field images. This approach made the architectures learn all weed species equitably, aligning optimisation with field requirements of detecting any weed present on farms, regardless of training frequency.

The epoch-level monitoring of F1-scores, without class discrimination, is similar with standard practice in pest and disease studies, where the F1-score metrics at each epoch is treated equally for all classes. This is because in agricultural settings weeds, pests, or pathogens may exhibit severe class imbalances, yet every class carries equal operational importance^45^.

For example, a farmer cannot accept to use a model that reliably detects common meadow grass but misses other major weed species like black grass, as this would be a cost to them. Moreover, farmers cannot accept a system that identifies wild oat while failing on creeping thistle or green foxtail. Consequently, the F1-score enforces this equitable performance requirement by treating each class as equally important in the optimisation objective, thereby preventing majority-class dominance from masking minority-class failures.

### Data analysis

We applied simple and multi-variable linear regression (MVLR) analysis, under the assumptions that model accuracy is linearly affected by occlusion, architecture, or both. This was done to compare the performance of ViTs and CNNs under occluded conditions, and whether occlusion during training improved accuracy. Consequently, two models were constructed:

1. Within-architecture analysis: Accuracy was regressed against occlusion level (random erasing *p* = 0,* 0.25*,* 0.5*) separately for CNN and ViT architectures, assessing whether increasing occlusion in training data improved resilience to field conditions.
2. Cross-architecture comparison: Model type (CNN vs. ViT) and occlusion level were included as joint predictors to isolate architecture-specific performance effects.

For each architecture, F-tests evaluated overall model significance, followed by two tailed t-tests on individual coefficients to determine whether occlusion level significantly influenced accuracy. To examine whether architectural differences yielded additional explanatory power beyond occlusion alone, partial F-tests compared full and reduced models.

This analysis quantified the extent to which ViT architectures contributed to accuracy gains independently of augmentation strategies, providing statistically grounded evidence for architecture-driven improvements in weed classification performance. It is noted that while statistical testing can be performed on the results obtained, dataset size can have an impact on the values produced, therefore our results are carefully considered under this fact.

## Results

### EfficientNet-B0 performance across varying occlusion levels

**Fig. 4:**
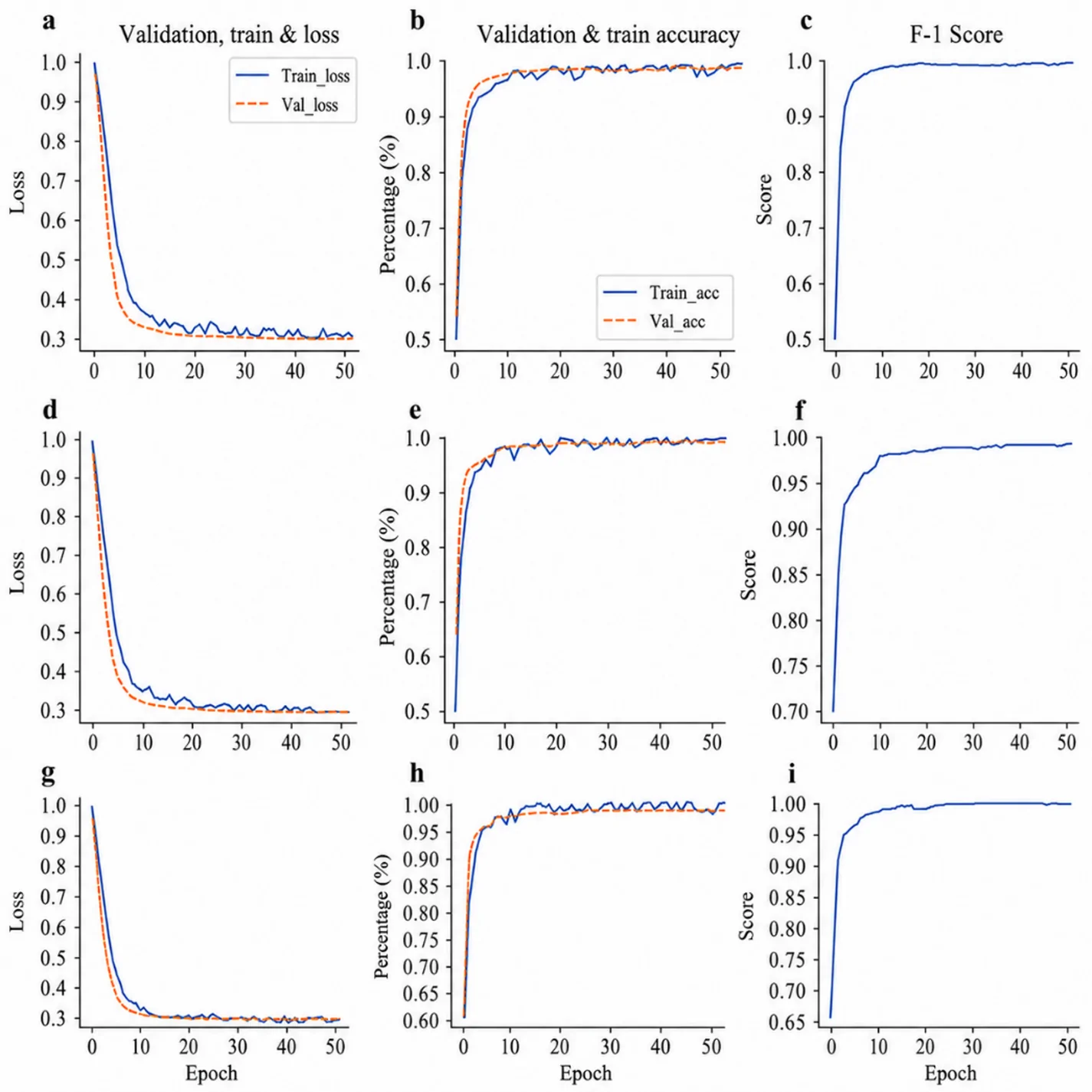
EfficientNet-B0 performance across various occlusion levels (a – i).

Our findings show that EfficientNet-B0 converged stably across all occlusion levels, but persistent validation oscillations indicated moderate overfitting. Without occlusion (0%), training and validation losses stabilised around 0.3–0.35 (Fig. 4a), with both accuracies reaching 99% (Fig. 4b) and F1-scores of 0.98 (Fig. 4c). Despite this, the test accuracy was only 65–68%, indicating that the model relied substantially on memorised training features, with minor validation fluctuations present. Introducing moderate occlusion (25%) maintained low loss values (Fig. 4d–f) and decreased testing accuracy to 53–54%, implying reduced distributed and generalisable feature learning. High occlusion (50%) further reduced testing accuracy to 45–47% (Fig. 4g–i), strengthening the implications of feature memorisation. While increasing occlusion reduced the overfitting gap and stabilised learning during training, residual validation instability suggests that EfficientNet-B0 remains partially constrained when classifying images with substantial regions removed.

**Fig. 5:**
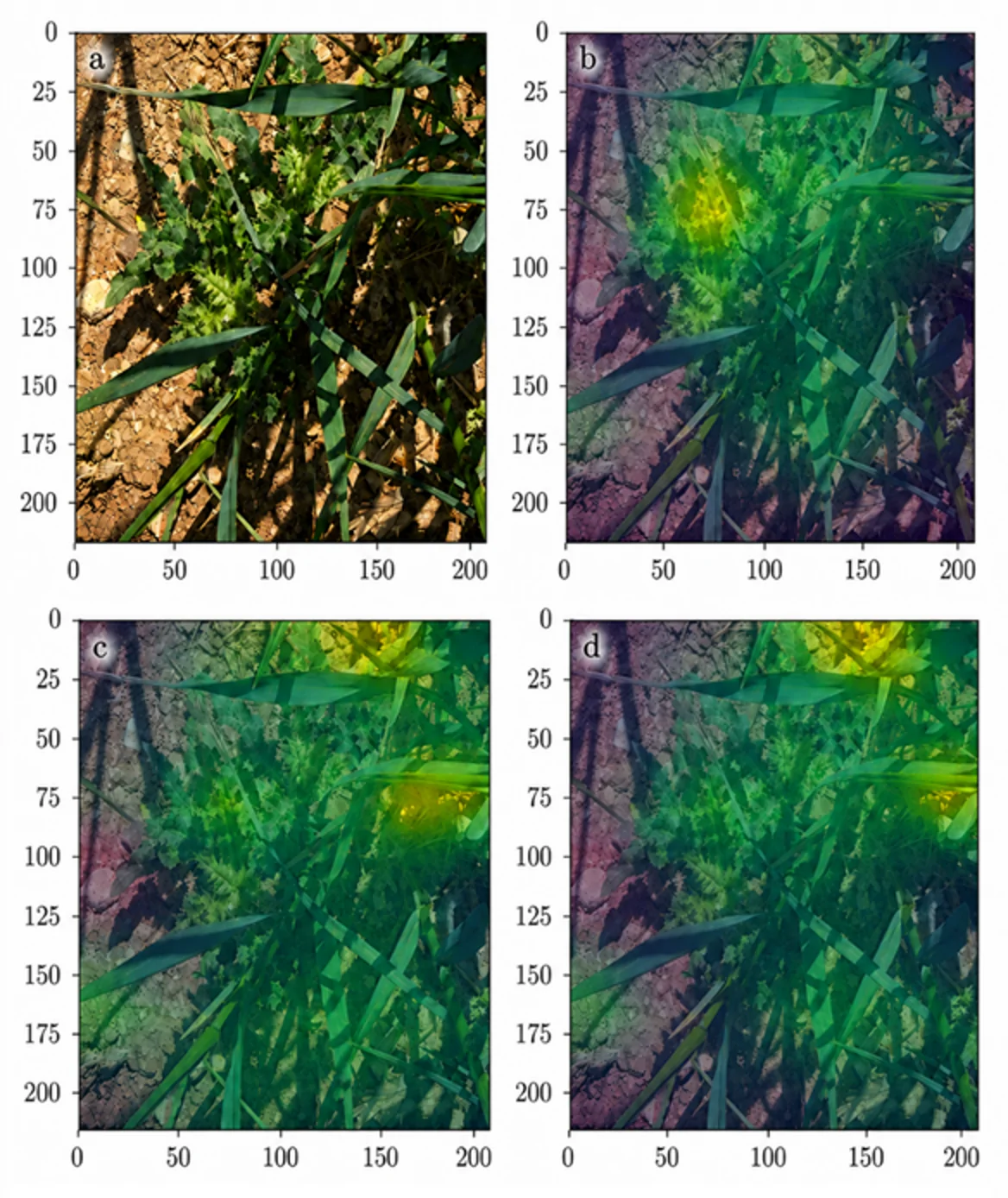
EfficientNet-B0 Feature Maps under varying levels of occlusion (**a**. Original image **b**. 0% occlusion **c**. 25% occlusion, **d**. 50% occlusion).

Feature attribution maps (Fig. 5a–d) show the representational basis of the accuracy gains under occlusion. At 0% occlusion, the model focussed on the visible parts of the target weed; as occlusion increased, attention spread to partially occluded target areas and, progressively, to surrounding non-target vegetation. The use of visual cues highlights the higher test accuracy, as the model learned to combine partial signals rather than rely on a single feature. However, the drift of attention onto neighbouring plants also suggests a weakness: in field conditions where intercrops and weeds grow closely together, the model may misclassify non-target plants as weeds.

### ResNet-50 performance across varying occlusion levels

**Fig. 6:**
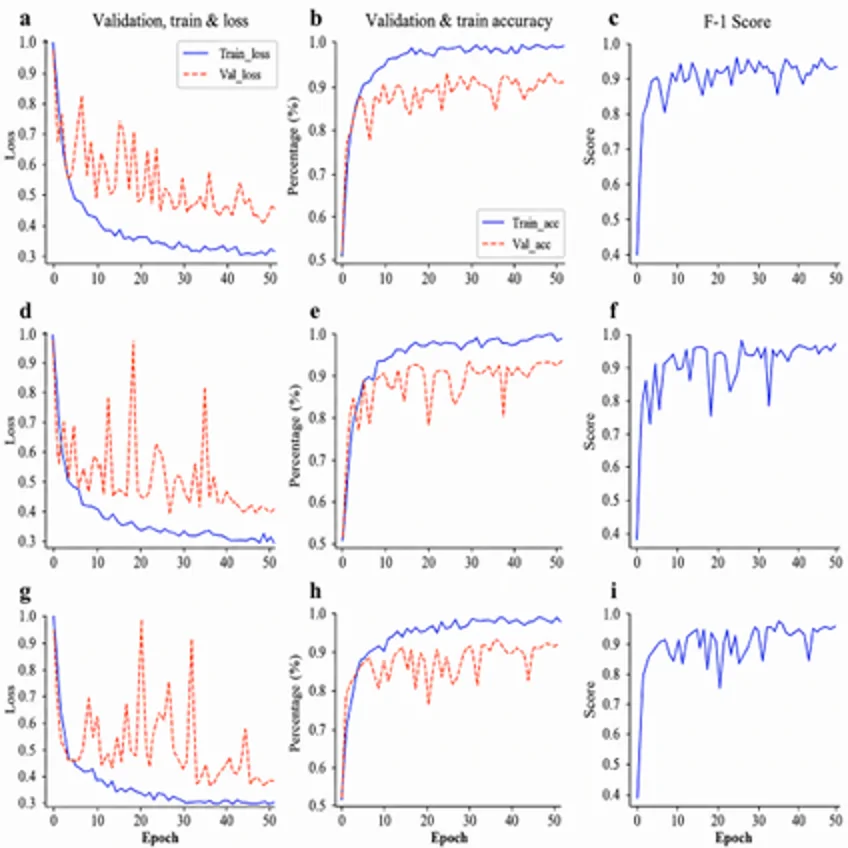
ResNet-50 performance across various occlusion levels (a – i).

ResNet-50 models showed substantial training instability and persistent validation oscillations across all conditions. On raw images, training loss decreased to 0.35 while validation loss stabilised between 0.40 and 0.50 with erratic oscillations (a), creating a 6–9% accuracy gap between training (99.3%) and validation (90–93%, Fig. 6b) despite F1-scores reaching 0.9 (Fig. 6c). Under 25% erasing, validation loss peaked near 0.4 at epoch 10 before stabilising at 0.3–0.35 (Fig. 6d), with no significant improvements in accuracy (99% training vs. 93% validation, Fig. 6e) and F1-scores of 0.9 (Fig. 6f). At 50% erasing, training and validation losses settles at 0.3 and 0.4 respectively (Fig. 6g), accuracies converged to 99.8% and 93% (Fig. 6h) and F1-scores reached 0.9 (Fig. 6i), and test accuracy was 76%.

**Fig. 7:**
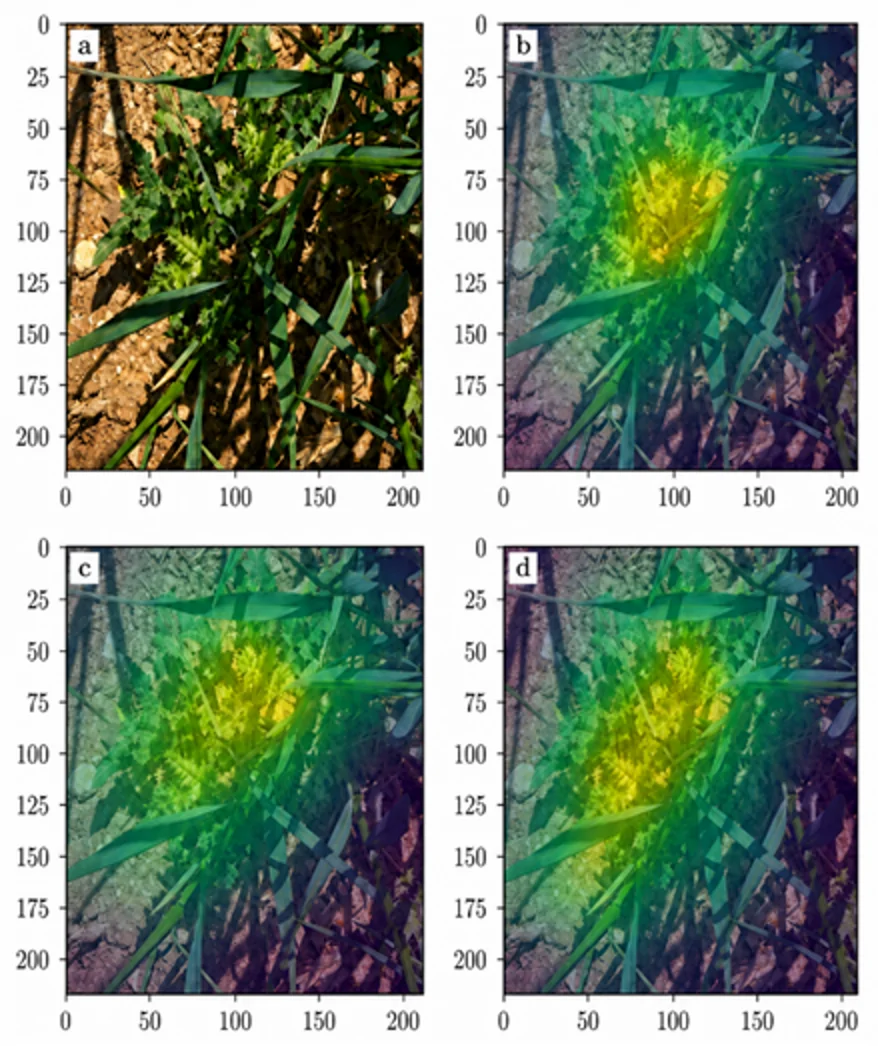
ResNet-50 Feature Maps under varying levels of occlusion (**a**. Original image **b**. 0% occlusion **c**. 25% occlusion, **d**. 50% occlusion).

Although the training–validation gap widened by only ~ 1% with increasing occlusion, the persistent oscillations indicate that ResNet-50 was less effective at handling the simulated occlusion patterns evaluated. This can be observed in Fig. 7a – d by the focus of the feature maps diffusing as the occlusion is increased, suggesting that the model is learning features not likely to be the plant itself.

### ViT-B16 performance across varying occlusion levels

**Fig. 8:**
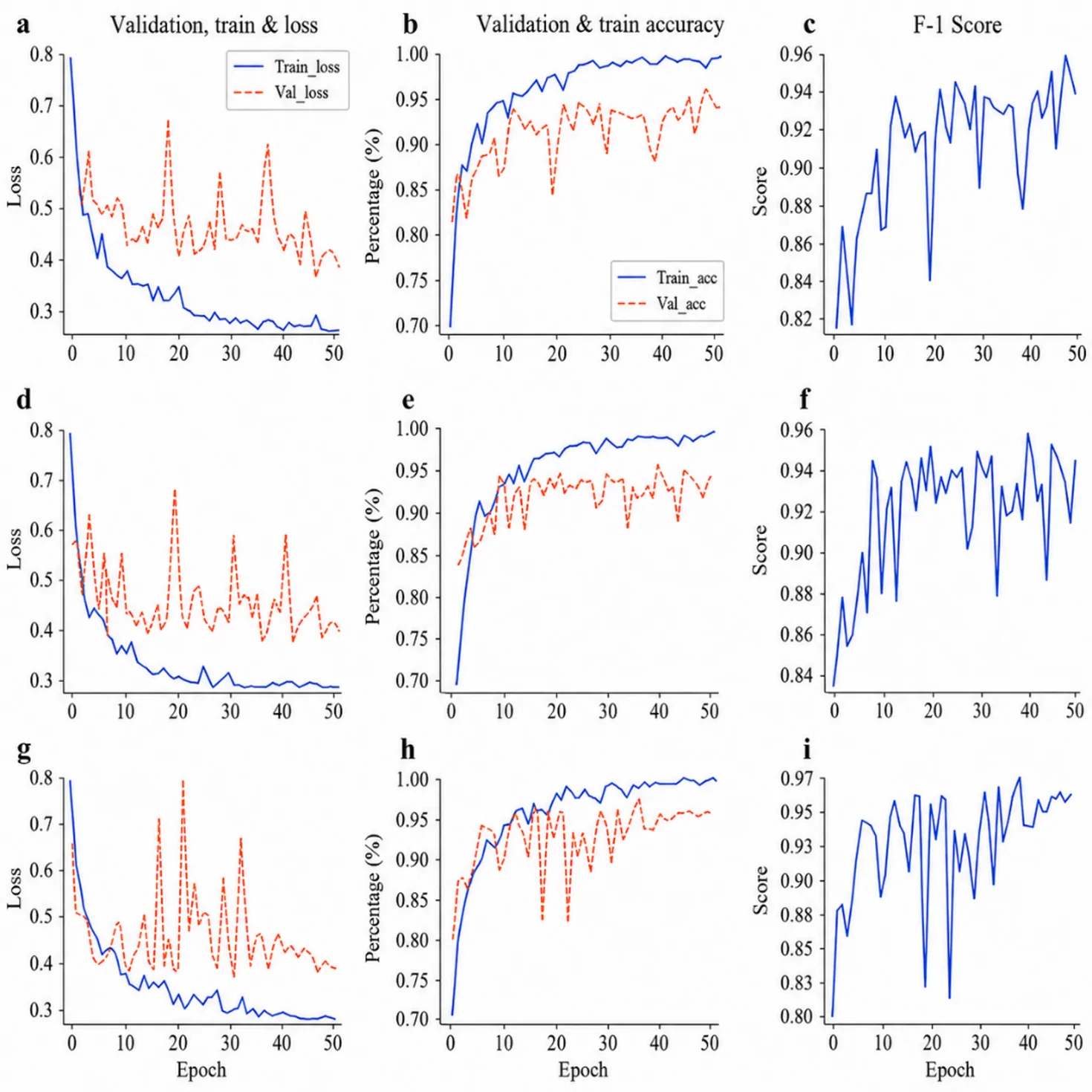
ViT-B16 performance across various occlusion levels (a – i).

ViT-B16 models achieved the highest classification accuracy despite severe training instability across all occlusion levels (Fig. 8b, h & g). Without occlusion (0%), training loss decreased to ~ 0.3 while validation loss stabilised between 0.45 and 0.5 (Fig. 8a). Training accuracy increased from 95 to 96% to near 100%, while validation accuracy was between 93 and 95% (Fig. 8b), and the F1-scores ranged from 0.82 to 0.96 (Fig. 8c). Testing accuracy was ≥ 80%, showing moderate overfitting despite strong internal performance. Under moderate occlusion (25%), convergence trends were similar (Fig. 8d – f), though the F1-score stability improved slightly (0.88–0.96) and testing accuracy increased to 84%. High occlusion (50%) introduced greater instability (Fig. 8g) but improved validation accuracy (94–96%, Fig. 8h) and F1-scores (0.80 to 0.96, Fig. 8i). Testing accuracy peaked at 86% (Table 4, a 6-point gain relative to the baseline).

**Fig. 9:**
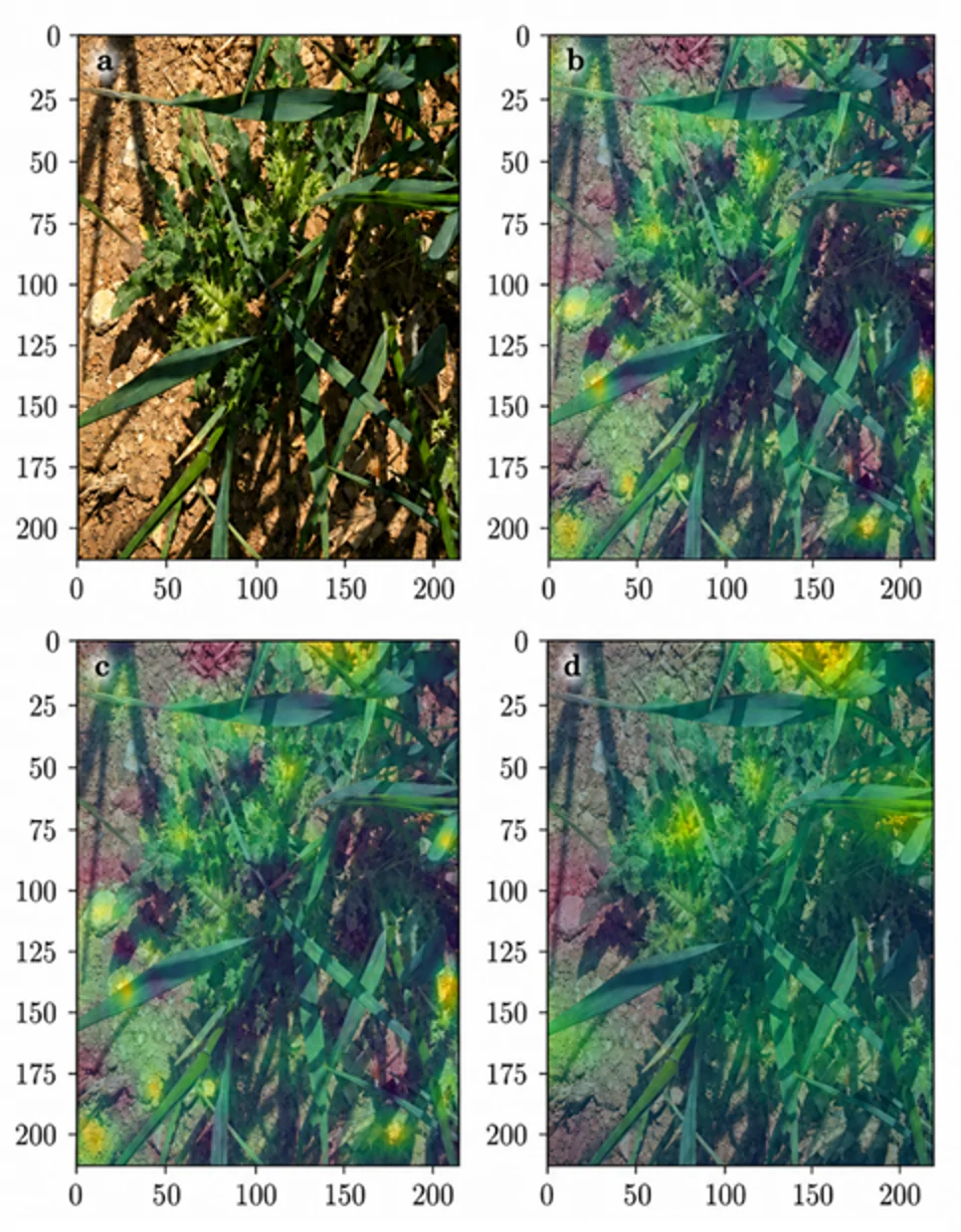
ViT-B16 attention maps under varying levels of occlusion (**a**. Original image **b**. 0% occlusion **c**. 25% occlusion, **d**. 50% occlusion).

Regarding the attention maps for ViT-B16, we found that the model increased its attention on hidden parts of the target weed as occlusion presence increased (Fig. 9d). However, it diverged its focus on more visible features present (Fig. 9b – c), where both plots show the attention over a greater area of more visible features of the target weed. On the other hand, Fig. 9d showcases a reduced disruption of focus compared to the dispersed attention of the remaining plots, reinforcing the increase in validation and testing accuracies as occlusion is increased.

### PvTv2 performance across varying occlusion levels

**Fig. 10:**
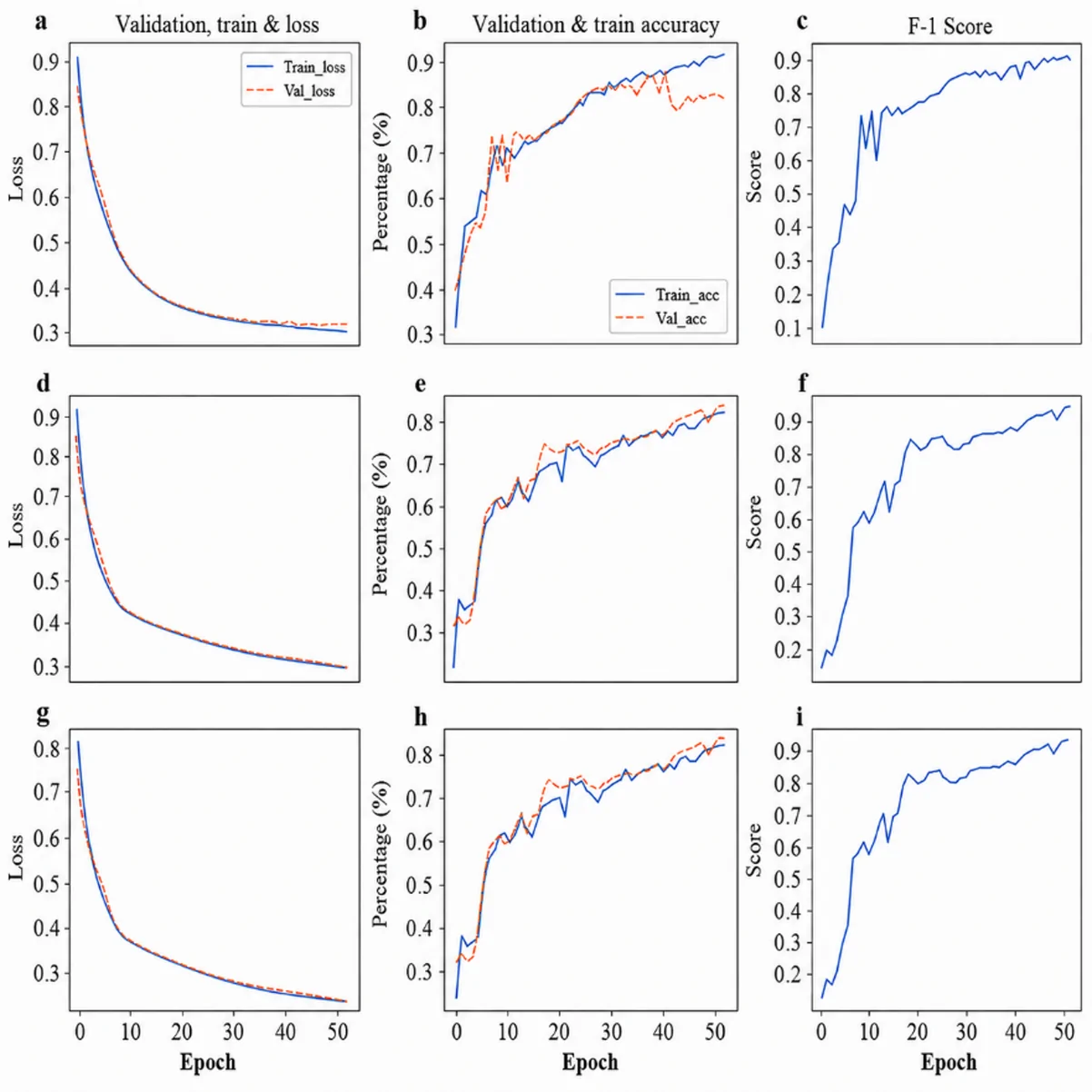
PvTv2 performance across various occlusion levels (a – i).

PvTv2 models demonstrated superior training stability across all occlusion levels, with training and validation metrics remaining closely aligned throughout training. Without occlusion (0%), both training and validation losses decreased from 6.0 to 0.2–0.4. and remained tightly coupled through epoch 50 (Fig. 10a), though training accuracy reached 93–95% while validation stabilised at 83–85% (Fig. 10b), indicating mild performance separation. F1-scores increased steadily from 0.1 to 0.9 (Fig. 10c), highlighting consistent learning progression. Moderate occlusion (25%) produced training and validation losses decreasing from 6.0 to 0.5–0.7 with perfect alignment (Fig. 10d), while training and validation accuracies converged tightly at 83–85% throughout training (Fig. 10e), eliminating the performance gap observed at 0% occlusion. F1-scores improved from 0.05 to 0.82–0.83 (Fig. 10f). High occlusion (50%) showed similar patterns with losses decreasing from 6.0 to 0.6–0.8 and remaining coupled (Fig. 10g), accuracies tightly aligned at 83–85% (Fig. 10h), and F1-scores reaching 0.82–0.84 (Fig. 10i). The non-plateaued loss trajectories and continued accuracy improvements across all occlusion levels suggest unrealised learning potential, indicating that PvTv2’s architecture is well-suited to occluded weed classification and may benefit from extended training schedules, particularly at higher occlusion levels where overfitting was eliminated.

**Fig. 11:**
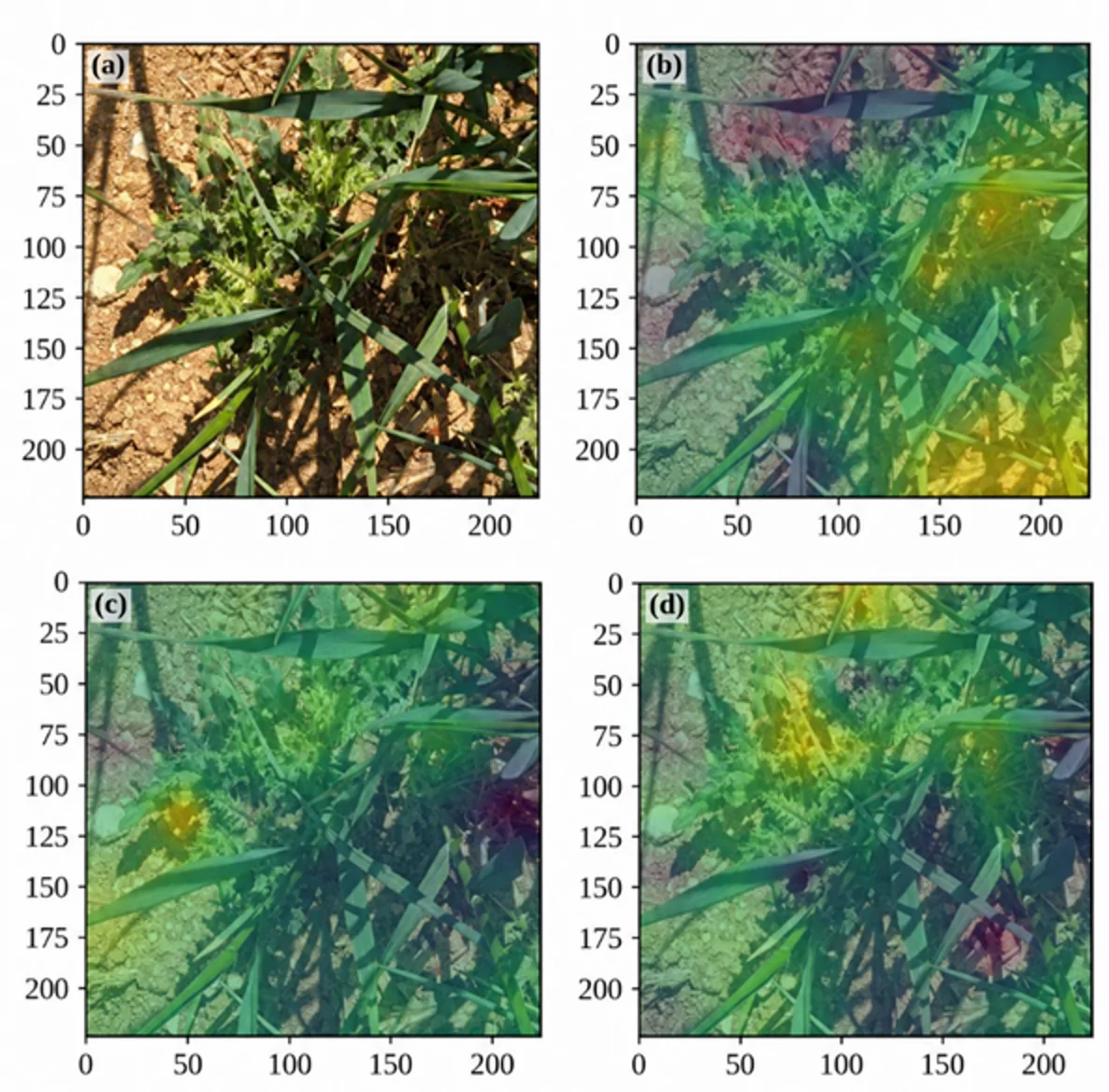
PvTv2 attention maps under varying levels of occlusion (**a**. Original image **b**. 0% occlusion **c**. 25% occlusion, **d**. 50% occlusion).

Observing PvTv2’s attention maps, its focus conversely ignores most of the target weed as synthetic occlusion is reduced or non-existent. As occlusion is increased, the model learns to focus its attention on more visible features (Fig. 11d) that are not as hidden as the attention in Fig. 11b. Moreover, PvTv2’s attention is concentrated and collated when compared to no occlusion present, which distributes itself in a more diluted manner. This implies that by increasing occlusion, the focus of the model can be shifted to behave in a more collected and consistent manner, reducing the drift of attention.

### Model test accuracy

**Fig. 12:**
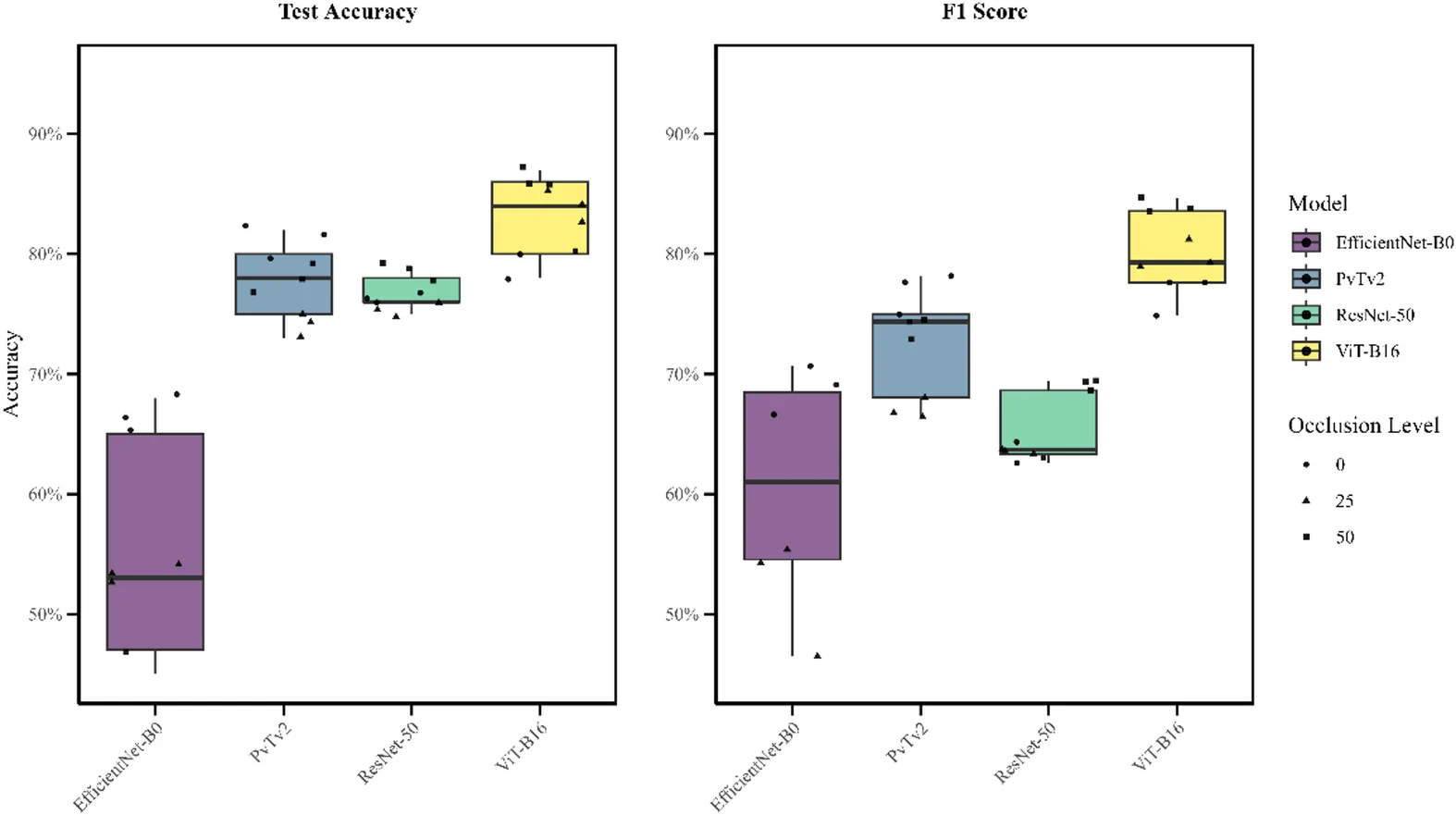
**a**) Test accuracy and b0 F1-score for each model under varying levels of occlusion.

To validate model performance, F1-scores were compared with test accuracy across the four architectures under increasing occlusion levels. Overall, the results show clear differences among the four architectures (Fig. 12a – b). Our findings reveal that EfficientNet-B0 recorded the lowest F1-scores, ranging from approximately 46–70%. Its performance declined with increasing occlusion, from around 67–70% at 0% occlusion to 46–55% at 25% and 41–43% at 50%. This pattern was consistent with its lower test accuracy, indicating that EfficientNet-B0 was highly sensitive to image obstruction. ResNet-50 showed moderate but relatively stable performance, with F1-scores ranging from approximately 62–69% across occlusion levels. Its test accuracy also remained stable, ranging from 75 to 79%. This suggests that ResNet-50 was less affected by occlusion than EfficientNet-B0, although its overall F1-score remained below that of the transformer-based models.

Regarding PvTv2, the architecture achieved intermediate performance, with F1-scores ranging from approximately 67–78% across the occlusion treatments. At 0% occlusion, PvTv2 recorded its strongest performance, achieving a test accuracy of approximately 82% and F1-scores of around 76–78%. Under 25% occlusion, performance declined, with test accuracy falling to approximately 75% and F1-scores reducing to around 67–70%, indicating that partial occlusion weakened class-level prediction. At 50% occlusion, test accuracy showed a slight recovery to approximately 77%, while F1-scores remained below baseline at around 70–72%. This suggests that although PvTv2 retained relatively balanced class-level performance compared with the CNN models, its robustness was reduced when image features were occluded.

ViT-B16 produced the highest F1-scores, increasing from approximately 75% at 0% occlusion to around 84–85% at 25–50% occlusion. This was consistent with its test accuracy, which increased from 80% to 86% as occlusion increased. These results indicate that ViT-B16 was the most robust architecture under partially occluded image conditions.

### Statistical influence of occlusion and architecture on model accuracy

To test whether the observed changes in accuracy were explained by occlusion severity or by model architecture, we fitted simple linear regressions and a MVLR model across all the architectures. We found that occlusion was a significant predictor of accuracy for EfficientNet-B0 and ViT-B16, but not for ResNet-50 or PvTv2 (Table 1). EfficientNet-B0 achieved the strongest occlusion-accuracy relationship, in which an increase in occlusion led to a negatively significant (*β* = −0.41, *p* ≤ 0.001) drop in its ability to distinguish weeds from the wheat crop. ViT-B16 showed an inverse similar pattern (*β* = 0.14, *p* ≤ 0.001, *R²* = 0.887), where an increase in occlusion improved its accuracy in distinguishing weeds from the wheat crop, while ResNet-50 and PvTv2 architectures produced very weak occlusion-accuracy relationships. Overall, these results indicate that occlusion does exert significant effects on some architectures, and that the direction and magnitude of the trend is contingent on the underlying architecture.

**Table 1:** Simple linear regression of occlusion over model accuracy.

Model (*N* = 9 per model)	Occlusion coefficient	*R*²	t-value	*p*-value
EfficientNet-B0	−0.41	0.962	−14.206	≤ 0.001
ResNet-50	0.05	0.334	2.24	0.06
ViT-B16	0.14	0.887	7.992	≤ 0.001
PvTv2	−0.07	0.515	−1.283	0.24

The multivariable regression results in Table 2 indicate that the testing accuracy of each model was driven primarily by architecture type rather than by occlusion level. Significant differences were observed (*p* ≤ 0.001) in which ViT-based architectures outperformed CNN-based architectures by an average of 14.56% points. Occlusion, by contrast, contributed no detectable independent effect once architecture was accounted for (*β* = −0.073, *t* = − 0.939, *p* = 0.354). These results underscore that architectural design is the primary driver of model performance under visually obstructed conditions.

**Table 2:** Multivariable regression results of occlusion and architecture types.

Variable (*N* = 18 per architecture)	Coefficient (β)	t-value	*p*-value
Architecture (ViT vs. CNN)	14.56	4.567	≤ 0.001
Occlusion	−0.073	−0.939	0.354

### Comparative model performance

**Fig. 13:**
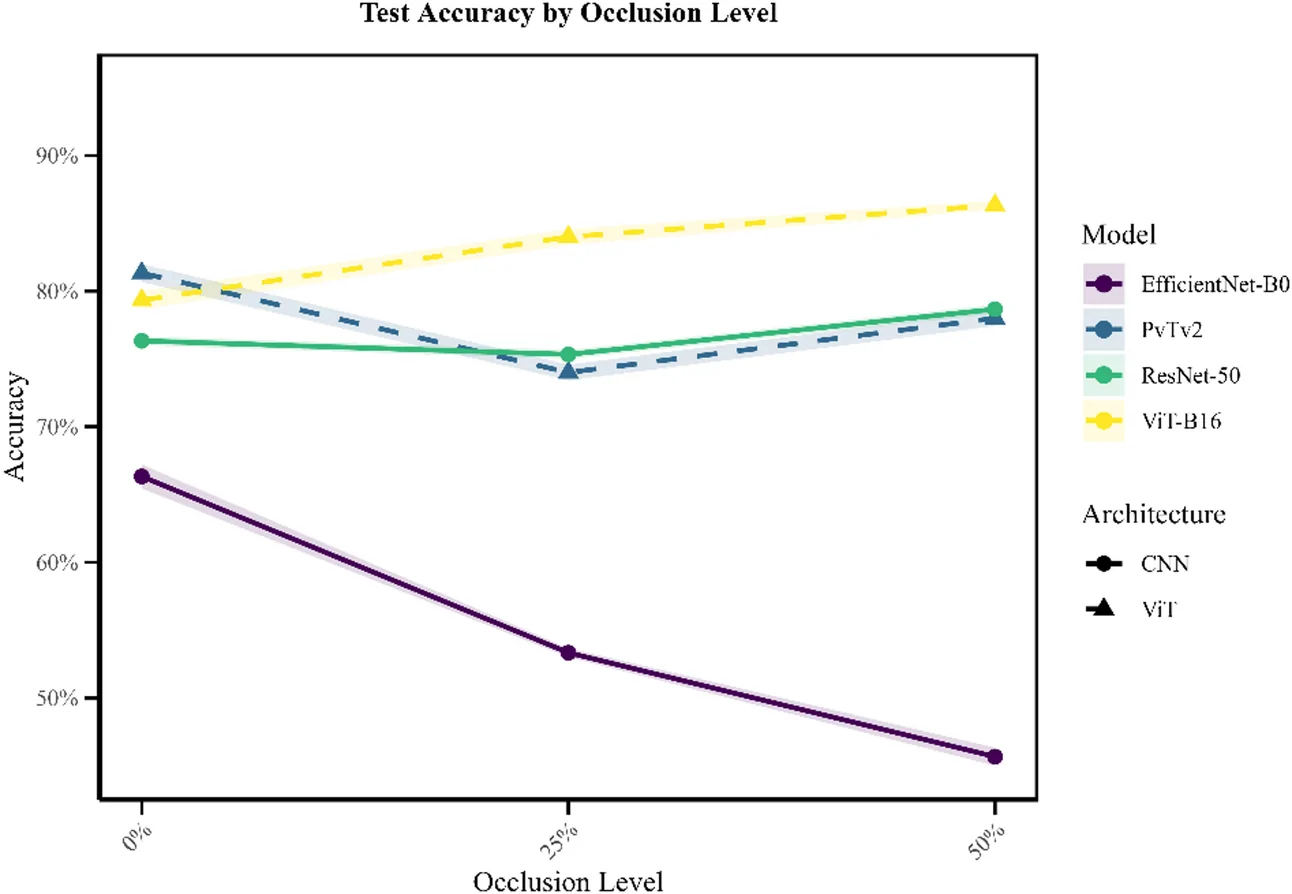
Mean test accuracy of models plotted over varying levels of occlusion.

Our overall findings show distinct performance patterns across the four architectures under increasing occlusion (Fig. 13). ViT-B16 demonstrated the strongest and most robust performance, with test accuracy increasing from approximately 80% at 0% occlusion to 84% at 25% occlusion and 86% at 50% occlusion. This suggests that ViT-B16 was resilient to partially obscured imagery and may have benefited from occlusion-based regularisation. ResNet-50 showed moderate but stable performance, with test accuracy remaining around 76% at 0% occlusion, 75% at 25% occlusion, and increasing slightly to 79% at 50% occlusion. In contrast, EfficientNet-B0 was the least robust architecture, with accuracy declining markedly from approximately 66% at 0% occlusion to 53% at 25% occlusion and 46% at 50% occlusion, indicating strong sensitivity to image obstruction. Interestingly, PvTv2 achieved the highest baseline accuracy, reaching approximately 82% at 0% occlusion. However, its accuracy declined to around 74–75% at 25% occlusion, before partially recovering to approximately 78% at 50% occlusion. This indicates that PvTv2 was affected by moderate occlusion but retained some predictive capacity under heavier occlusion. These contrasting responses highlight the importance of architectural design in handling occluded weed imagery under realistic field conditions, with ViT-B16 emerging as the most robust model and EfficientNet-B0 as the most sensitive to occlusion.

### Computational profile and architectural confounds

To contextualise the performance differences observed across architectures, we profiled each model’s computational cost, parameter count, and inference time on a uniform hardware platform (16 GB DDR4 RAM, Nvidia GTX 1660 Super GPU) (Tables 2 and 3). There were two orders of magnitude for parameter counts, ranging from 0.34 million (M) for PvTv2 to 23.54 M for ResNet-50, with ViT-B16 (8.58 M) and EfficientNet-B0 (4.01 M) occupying intermediate positions. Occlusion robustness did not scale with model size such that the smallest model (PvTv2) showed the steepest performance degradation under occlusion, while the largest (ResNet-50) was outperformed by the substantially smaller ViT-B16. This uneven relationship between model size and robustness indicates that representational capacity alone does not account for the observed differences in occlusion robustness.

Computational cost varied more with performance. ViT-B16 required 16.74 GFLOPs per image, approximately twice that of EfficientNet-B0 (8.39 GFLOPs) and five times that of ResNet-50 (3.32 GFLOPs) and PvTv2 (3.50 GFLOPs). Mean inference time followed the same pattern (17.06 s (s) for ViT-B16 versus 3.44s, 3.22s and 1.82s for EfficientNet-B0, ResNet-50 and PvTv2 respectively), reflecting the quadratic complexity of full self-attention over the input patch sequence. ViT-B16’s superior occlusion robustness therefore co-occurs with substantially greater per-image computation, and the present experimental design does not disentangle the contribution of architectural inductive bias from that of compute budget. PvTv2, despite sharing a transformer foundation with ViT-B16, operated at a fraction of the computational cost (3.50 GFLOPs, 1.82s) and produced the weakest occlusion performance, consistent with its hierarchical attention design trading global receptive field for efficiency.

**Table 3:** Inference time of each model.

Inference time (s) per occlusion level	CNNs		ViT	
	EfficientNet-B0	ResNet-50	ViT-B16	PvTv2
0%	8.39	3.32	16.74	3.5
25%	0.93	3.15	17.06	0.96
50%	1.01	3.2	13.38	0.99
Mean inference time	3.44	3.22	17.06	1.82
Per image batch	0.11	0.1	0.5	0.06
Per image	0.01	0.01	0.06	0.01

These computational metrics carry direct deployment implications. EfficientNet-B0 and PvTv2, with sub-second per-batch inference times and parameter counts under 5 M, are suited to resource-constrained settings such as edge devices in field robotics, where moderate occlusion robustness is acceptable. ViT-B16’s superior accuracy under occlusion comes at a cost-per-image more than nine times that of PvTv2, restricting its viability to server-side or offline inference workflows. The architectural choice for occlusion-robust classification is therefore not solely a question of accuracy but a trade-off between robustness and computational tractability that depends on the target deployment environment.

**Table 4:** FLOPs and parameter count of each model.

FLOPs	CNNs		ViT	
	EfficientNet-B0	ResNet-50	ViT-B16	PvTv2
GFLOPs (per image)	8.39	3.32	16.74	3.5
TFLOPs (total)	0.93	3.15	17.06	0.96
Parameter Count (Millions)	4.01	23.54	8.58	0.34

## Discussion

Our findings reveal that model architecture type influences learning and classification of images under varying degrees of occlusion. ViT‑B16 achieved high testing accuracy (≥ 80%) and stable F1‑scores (0.82–0.96) even when up to 50% of the image was naturally and synthetically hidden, showing robust feature extraction, recognition and resilience to visual disruption. In contrast, EfficientNet-B0 and ResNet-50 showed higher performance loss (training/validation) with increasing occlusion. At moderate occlusion (25%), both models showed slightly improved generalisation, however, both still showed unstable validation curves, with testing accuracy dropping below 60% under heavy occlusion (50%). PvTv2 achieved stable training validation overall, however, as occlusion increased, its accuracy declined, indicating reduced representation capacity for highly occluded inputs.

However, a key consideration when interpreting our findings is the challenge of attributing performance differences to individual architectural features. This is because the four models tested in our study differ in various ways such that each has multiple correlated dimensions and the fact that the current design compares these architectures jointly rather than independently. However, the non-uniform relationship between model size and occlusion robustness weakens the explanation that ViT-B16’s advantage is from greater representational capacity alone, while its substantially higher computational cost co-occurs with that advantage and cannot be separated from the contribution of self-attention in the current setup. We therefore interpret ViT-B16’s superior occlusion robustness as consistent with the global receptive field afforded by full self-attention, while acknowledging that fully controlled comparisons across architectures are required to isolate the specific causal contribution of any single design choice.

To contextualise the performance differences observed across architectures, we profiled each model’s computational cost, parameter count, and inference time on a uniform hardware platform (Tables 2 and 3). We found that the smallest model (PvTv2) exhibited the steepest degradation under occlusion, whereas the largest (ResNet-50) was outperformed by the substantially smaller ViT-B16. This uneven relationship confirms that representational capacity alone does not explain the observed differences in occlusion robustness, extending findings by Tan and Le^46^, who showed that compound scaling in CNNs improves accuracy but did not examine occlusion. This is also consistent with Sanderson and Matuszewski^47^, who reported that architectural inductive bias and parameter count do not solely determine visual reasoning quality.

We have also found that computational cost varies substantially across architectures. ViT-B16 required larger GFLOPs per image, which is more compared to the other architectures in this study. The mean inference time followed the same pattern, reflecting the quadratic complexity of full self-attention over the input patch sequence. These metrics align with the efficiency analyses of Jamil et al.^48^, who argue that ViTs self-attention scales quadratically with sequence length. Our findings consequently extend their results to weed identification and classification under controlled occlusion. ViT-B16’s superior occlusion robustness therefore co-occurs with substantially greater per-image computation, and the present experimental design does not disentangle the contribution of architectural inductive bias from that of compute budget. PvTv2, despite sharing a transformer foundation with ViT-B16, operated at a fraction of the computational cost and produced the weakest occlusion performance, consistent with its hierarchical attention design trading global receptive field for efficiency. This reveals that pyramid vision transformers lose long-range dependency modelling for computational efficiency^49^, but we uniquely quantify this cost in terms of occlusion-specific accuracy degradation across wheat weed species.

These computational metrics carry direct deployment implications. EfficientNet-B0 and PvTv2, with sub-second per-batch inference times and parameter counts under 5 M, are suited to resource-constrained settings such as edge devices in field robotics, where moderate occlusion robustness is acceptable. ViT-B16’s superior accuracy comes at a cost-per-image, which is more than nine times that of PvTv2, restricting its viability to server-side or offline inference workflows. The architectural choice for occlusion-robust classification is therefore not solely a question of accuracy but a trade-off between robustness and computational tractability that depends on the target deployment environment. As such, our findings show that for field settings with irregular, partial visibility, the robustness-efficiency trade-off fundamentally differs from natural image classification, necessitating architecture-specific deployment strategies rather than generic efficiency optimisation.

Within this caveat, the differential performance we observed is consistent with fundamental architectural differences between CNNs and ViTs. As Ji et al.^50^ demonstrated that the limited receptive field of CNNs constrains their ability to model long-range dependencies, whereas ViTs split images into patches and apply a token-based self-attention mechanism that explicitly captures global context. This mechanism enables ViTs to collect information across distant regions of an image, enabling reconstruction of partially hidden weeds, while CNNs struggle once key visual information is hidden. Such architectural distinctions carry practical implications for agricultural settings where occluded, cluttered, or partially visible weed and crop features are common. The occlusion tolerance of ViTs is particularly relevant to the development of spot-spraying and autonomous weed, disease and/or pest management systems^51^, where classification reliability under partial visibility determines precise chemical application and crop protection efficacy^52^.

A more granular analysis highlights how architecture type influences representation learning under occlusion. This is because the quality of extracted features influences classification performance^53^. In our study EfficientNet-B0 improved generalisation under moderate occlusion (25%) but declined sharply beyond 50% occlusion. Similarly, ResNet-50, despite its greater depth enabling smoother gradient flow, displayed persistent validation oscillations, confirming that deeper depth alone cannot offset missing spatial information when receptive fields remain local. While Mukti et al.^54^ suggests that hierarchical CNNs with multi-scale fusion can partially mitigate these limitations, their findings were limited to structured medical imaging settings with structured occlusion rather than the irregular, context-rich occlusion found in agricultural imagery. In addition, the oscillation within these two CNN architectures indicates that increased network depth without global feature aggregation provides limited resilience to partial occlusion, as the model struggles to reconstruct missing spatial information from local features alone. Recent transformer variants such as modified MaxViT, MobileViT and hybrid TransXAI achieved up to 100% acuracy on crop‑disease tasks^55^, outperforming DenseNet-161 and MobileNetv2 and demonstrating that self-attention-based models offer higher classification accuracy and robustness than CNN.

We also observed that PvTv2 maintained excellent training stability, but showed the largest accuracy decline under increasing occlusion. This underscores a critical insight that training stability alone does not guarantee operational robustness. Notably, even under 50% occlusion, ViT-B16’s distinction between architectural stability during training and feature extraction capability is commendable even when critical image regions are absent^56^. This finding emphasises that stable training dynamics do not necessarily translate to effective performance under difficult field conditions. As a result, even at 50% occlusion, ViT-B16 outperformed PvTv2 by ~ 10%, and the CNN models by ~ 8%, confirming the practical value of transformer-based global feature aggregation^57^.

Our findings extend studies that show that the self-attention mechanism in ViT-B16 treats an image as a sequence of patches and learns relationships across the entire frame, enabling the network to capture long-range dependencies that CNNs miss^58^. This architectural advantage is valuable in farm settings where weeds are routinely hidden. Interestingly, Zeineldin et al.^59^ have reported that the limited receptive field of CNNs constrains their ability to model global context, whereas ViTs overcome this by explicitly capturing global context via token-based self-attention.

However, Chaman and Dokmanic^60^ have challenged this perspective, arguing that sufficiently deep CNNs can approximate global receptive fields while maintaining computational efficiency. Conversely, their findings are based on natural image datasets rather than agricultural scenarios with varying levels of occlusion, limiting their applicability to our context. As a result, we argue that the model’s balanced precision–recall trade-off, reflected in consistent F1 scores across occlusion levels, is especially critical for field deployment. This is because prediction instability can compromise herbicide application accuracy, leading to either missed weed targets (false negatives) or blanket herbicide application to crop areas (false positives), both of which undermine the efficacy and sustainability of precision spraying systems.

### Comparisons with other studies

Comparing our findings with existing studies reveals both convergences and critical divergences in how occlusion robustness is addressed across architectures for weed classification. Our findings suggest that ViT based architectures offer meaningful advantages over CNNs for weed detection under occlusion. We show that ViT-B16 has properties not observed in convolutional baselines: rather than degrading, accuracy increased as synthetic occlusion rose to 50%. We interpret this as a consequence of global self-attention, which aggregates dispersed contextual cues across the whole image, whereas the localised receptive fields of convolutional models appear more vulnerable when discriminative regions are hidden.

Direct comparison of our findings with other studies is possible but complicated due to differing tasks, datasets, and hardware. For example, Wang et al.^61^ in their study found higher predictive accuracy in weed detection with CNN architectures, but did not evaluate the impact of occlusion. Islam et al.^62^ prioritised efficient inference yet evaluated a single weed species and reported reduced crop (soybean and corn) detection. Furthermore, Rai and Sun^63^, achieved a higher unified weed detection–segmentation but experienced on-device degradation and did not address partial visibility. These studies advanced diversity, speed, and task unification respectively, dimensions our work does not cover. However, we extend their work by comparing occlusion robustness between ViTs and CNNs. We therefore frame our results as evidence that global attention is a promising direction for occlusion-resilient detection, while acknowledging that edge-deployment feasibility remains an important and unaddressed limitation of the present study.

**Table 5:** Performance comparison of computer vision architectures.

Aspect	This study	Rai & Sun^63^	Islam et al.^62^	Wang et al.^61^
Focus	Occlusion robustness comparison	Single-stage detection + instance segmentation	Lightweight edge deployment for real-time weed detection	Multi-class weed dataset
Models evaluated	ViTs (ViT-B16, PvTv2) and CNNs (EfficientNet-B0, ResNet-50)	Custom YOLO (C3/C3x modules, ProtoNet)	SSD-MobileNetV3, DeepLabv3+-MobileNetV3, YOLOv8n, MobileNetV4-Seg	YOLOv3, YOLOv5, Faster R-CNN
Dataset size	970 original weed images (3 species)	767 original to 2,257 − 2,685 augmented (3 species)	480 − 443 sub-images ( 3 species)	14,035 images (25 species)
Performance	ViT-B16: ~86% accuracy at 50% occlusion; PvTv2: ~82% (0% occlusion) to ~ 78% (50% occlusion)	C4 model: 85.4% (bbox precision), 82.8% (mask precision)	MobileNetV4-Seg: 69.9% IoU (corn), 76.8% IoU (soybean), 44 FPS	YOLOv5: 92.4% mAP; Faster R-CNN: 92.15% mAP
Occlusion handling	Systematically tested (0%, 25%, 50%)	Not quantified	Not quantified	Not quantified
Edge deployment	Not implemented	TorchScript & ONNX on Jetson AGX Xavier	Jetson Nano (33 FPS SSD) & Jetson Orin Nano (44 FPS)	Desktop GPU only
Computational cost	ViT-B16: 16.74 GFLOPs, 17.06s inference	0.34 M − 23.54 M parameters; 0.11–0.5 s per batch	MobileNetV4-Seg: 1.93 M parameters; SSD: 33 FPS	Not reported
Key strength	Occlusion tolerance mechanism identified (self-attention vs. local receptive fields)	Combines detection + instance segmentation in single stage	Real-time edge inference (44 FPS) with segmentation	Largest species diversity (25 classes)
Key limitation	High computational cost; small dataset (970 images)	Limited species (3); no occlusion testing	Single weed species; limited field variability	No occlusion testing; no edge deployment

### Study implications

These results carry direct implications for site-specific herbicide application and sustainable weed management. Robust classification under occlusion enables precise, targeted spraying and reduces blanket herbicide applications, which helps limit selection pressure for resistance in weed population^64^. Moreover, consistent classification across variable field conditions reduces off-target contamination of crops, soil, and water systems. Early and accurate identification of weeds even under partial visibility supports earlier intervention when crops are more susceptible to control and before substantial yield losses occur. Furthermore, the enhanced robustness observed in ViT-based architectures suggests that future precision agriculture systems can maintain high performance standards in less-than-ideal field environments, potentially increasing the adoption rate of autonomous weeding robots.

Our results align with emerging evidence from agricultural imaging demonstrating the superiority of self-attention architectures under partial visibility conditions. Recent studies in crop disease classification^65^, fruit recognition^66^, and plant phenotyping^67^ have similarly reported enhanced robustness of transformer-based models compared to CNNs when dealing with occlusion, variable lighting, and complex background conditions. By extending these findings to occlusion-challenged weed classification, our study provides one of the first systematic demonstrations of how architectural choice directly affects deployable performance in precision agriculture. Ultimately, our study advocates for a paradigm shift in model selection for agricultural AI, where robustness to field-realistic occlusions rather than just baseline accuracy is prioritised as a critical metric for long-term viability in complex outdoor settings.

### Limitations

This study is subject to several limitations. Our dataset, though designed to simulate occlusion scenarios, was augmented to increase sample size, which may introduce bias, data leakage, and reduce representativeness during field deployment. In addition, computational constraints limited the training duration and complexity of some models, as well as the overall number of statistical tests. Increased computational power could provide more significant results by allowing a greater number of comparative tests to be completed from the trained models. Moreover. while PvTv2 could be more likely to run on local systems due to its parameter and FLOP count (0.34 million), cloud deployment is more suited, providing a barrier to farms with little to no internet. Additionally, the synthetic nature of the occlusion applied in our study, while useful for establishing a controlled baseline, may not fully capture the complex, non-uniform shapes of natural weed-crop occlusions found in high-density agricultural fields.

Future research should extend to larger, multi-seasonal, multi-location field datasets and incorporate temporal sequences (e.g., successive passes of UAV or ground vehicles) to further test model resilience under dynamic conditions. Hybrid CNN–Transformer architectures that combine spatial efficiency with global context might offer scalable, edge-deployable solutions for real-time precision-agriculture weed classification. Consequently, validating these findings in real-time, on-device field trials remains a necessary step to bridge the gap between architectural robustness and practical implementation.

## Conclusion

Our work presents advancements in understanding architectural influences on weed classification performance in PA. This research indicates that the inherent differences in feature extraction mechanisms between ViTs and CNNs fundamentally influence their behaviour to occluded images in agricultural applications. In this work, the ViT-B16 model proved it’s resilience, achieving stable, high-accuracy performance even when visual evidence was partially occluded by crop canopy, soil debris, or overlapping vegetation. This superiority stems from its global attention mechanism, which allows the model to leverage non-local contextual information for robust feature integration. This capability inherently overcomes the limitations of CNNs’ local receptive fields when dealing with occluded or partially visible weed specimens in complex field scenarios.

The architectural advantages identified in this study are relevant to precision agriculture applications where classification reliability under partial visibility is critical, including drone-based site-specific spraying systems and herbicide resistance management. Enhanced classification accuracy under occlusion has the potential to support more targeted herbicide application, with downstream benefits for reduced chemical usage and environmental impact. However, the present study was conducted on a curated image dataset under controlled synthetic occlusion, and these practical benefits remain prospective until validated through field trials on operational platforms and replication on larger, more diverse datasets capturing the full range occlusion sources. By providing a clear framework for evaluating how model architecture affects robustness, our research establishes a foundational step toward more reliable, intelligent robotic systems capable of navigating the unpredictable visual environments inherent to modern field agriculture.

Future work should therefore prioritise deployment trials with autonomous spraying systems and benchmark performance against current commercial weed detection solutions to establish whether the architectural advantages observed here translate into measurable agronomic and environmental gains in the field. As a result, this study reinforces the necessity of prioritising model robustness as a key performance indicator, bridging the gap between theoretical computer vision advancements and the practical demands of sustainable, large-scale weed management.

## Data Availability

The datasets generated and/or analysed during the current study are publicly available on the link here https://drive.google.com/file/d/1YnGaE4E-FTTx8j8VqcGFqSN6AOSlxv10/view. The reproducible code can be accessed on the following link https://github.com/Euan-Hall/Masters-Code.
